# Navigating Academic Identity: A Qualitative Exploration of Graduate Students’ Conference Participation Behaviors and Academic Socialization Processes

**DOI:** 10.3390/bs16020217

**Published:** 2026-02-03

**Authors:** Mengting Qian, Zeqing Xu, Chunshun Yan

**Affiliations:** 1 Faculty of Education, East China Normal University, 3663 North Zhongshan Road, Putuo District, Shanghai 200062, China; 52264110004@stu.ecnu.edu.cn (Z.X.); chunshun.yan@mail.utoronto.ca (C.Y.); 2 Berkeley School of Education, University of California, Berkeley, 2121 Berkeley Way, Berkeley, CA 94720, USA; 3 Ontario Institute for Studies in Education, University of Toronto, 252 Bloor Street West, Toronto, ON M5S 1V6, Canada

**Keywords:** academic socialization, graduate students, academic conferences, professional identity, academic alienation, qualitative research

## Abstract

This qualitative study investigates how graduate students engage with academic conferences as sites for professional development and identity construction. Grounded in academic socialization theory and employing an interpretive phenomenological approach, we conducted semi-structured interviews with 18 graduate students across diverse disciplinary fields in China. Reflexive thematic analysis revealed four distinct participation orientations: knowledge-seeking, competence-building, network-oriented, and identity-exploratory. Our findings illuminate how contemporary academic environments characterized by heightened competition, publish-or-perish pressures, and quantified evaluation systems create conditions of academic alienation, manifesting as disconnection from scholarly work, superficial collegial interactions, and weakened community belonging. Significantly, we identified conference participation as a transformative mechanism through which students counteract alienation by reclaiming meaning in scholarly labor, cultivating authentic academic dialogue, and reconstructing professional community ties. We propose an integrative conceptual framework illustrating the dynamic relationships among alienation, participation orientations, and transformative outcomes. These findings advance the theoretical understanding of academic socialization as an agentic, iterative process and offer practical implications for institutions, faculty advisors, and students seeking to support graduate student development in increasingly pressurized academic climates.

## 1. Introduction

Academic conferences constitute a distinctive arena within the landscape of scholarly practice, serving not merely as venues for knowledge dissemination but as consequential sites where emerging scholars negotiate their professional identities and cultivate disciplinary belonging (Collins et al., 2023; Lucia et al., 2025). For graduate students navigating the transition from novice to independent researcher, conference participation represents a critical yet understudied dimension of academic socialization—the complex process through which individuals acquire the knowledge, skills, values, and dispositions necessary for effective membership in scholarly communities (Weidman et al., 2001).

Graduate students today navigate an academic landscape increasingly defined by competition and performance metrics. Escalating publication demands, the expansion of quantitative evaluation criteria, and growing uncertainty in academic career pathways have fundamentally transformed the conditions shaping doctoral and master’s students’ scholarly development (Castelló et al., 2017; Mantai, 2017; SenthilKumar et al., 2023). Research shows that as many as one-third to one-half of graduate students report symptoms of depression, anxiety, or professional burnout during their training period (Satinsky et al., 2021; Evans et al., 2018; Levecque et al., 2017). These structural transformations raise important questions about how graduate students experience and make meaning of their academic work, and what resources and practices support their professional formation (Chi et al., 2023).

The “publish or perish” culture that pervades contemporary academia creates particular challenges for graduate students who must simultaneously develop research competencies and demonstrate productivity (Grimes et al., 2018; Heffernan, 2021). This pressure extends beyond traditional publishing to encompass conference participation, networking, and other forms of scholarly engagement that contribute to academic reputation and career advancement (Horta et al., 2024). Understanding how students navigate these demands—and what practices help them maintain connection to the intrinsic values of scholarship—has become increasingly urgent.

While existing scholarship has examined various dimensions of graduate student development—including mentoring relationships (Baker & Pifer, 2011), writing and publication experiences (Inouye & McAlpine, 2019), doctoral attrition (Gardner, 2009), and interdisciplinary socialization (Boden et al., 2011)—conference participation as a specific developmental context remains relatively unexplored. Previous studies have primarily adopted institutional or quantitative perspectives, examining participation rates, funding mechanisms, or organizational factors (Rowe, 2018). Less attention has been devoted to understanding how graduate students themselves experience conference engagement, what motivates their participation, and how these experiences contribute to their evolving academic identities.

This gap is significant because conferences offer unique affordances for academic socialization that differ from other developmental contexts. Unlike the hierarchical dynamics of advisor–student relationships or the solitary nature of dissertation writing, conferences situate graduate students within a broader scholarly community where they can observe disciplinary norms, receive feedback on their work, build professional networks, and envision future career trajectories (Nicolson, 2017; Shin et al., 2018; Wu et al., 2025). The intensive, time-bounded nature of conference experiences creates conditions for accelerated identity work and the crystallization of emerging self-understandings as scholars (Liddle, 2025). Moreover, virtual communities of practice that have emerged alongside traditional conferences offer additional opportunities for academic networking and mutual support (Hayes et al., 2024). Understanding how students navigate these experiences—particularly in contexts characterized by intensified competition—can inform efforts to support their development as scholars and professionals.

The present study addresses these gaps through an in-depth qualitative exploration of graduate students’ conference participation behaviors and experiences. Specifically, we investigate the following research questions: (1) What patterns characterize graduate students’ motivations for and approaches to academic conference participation? (2) How do students perceive the relationship between conference participation and broader academic environmental challenges? (3) In what ways does conference participation contribute to students’ academic socialization and identity development?

By examining these questions through the perspectives of graduate students themselves, this study contributes to the theoretical understanding of academic socialization as an agentic, contextually embedded process while offering practical insights for institutions, mentors, and students seeking to optimize developmental outcomes from conference engagement.

## 2. Literature Review

### 2.1. Graduate Student Development and Academic Socialization

Research on graduate student development has expanded significantly over the past two decades, driven by growing recognition of the unique challenges facing doctoral and master’s students in contemporary higher education (Gardner, 2009). Scholars have examined multiple dimensions of graduate experience, including mentoring relationships (Baker & Pifer, 2011), writing development (Inouye & McAlpine, 2019), program completion and attrition (Castelló et al., 2017), and career preparation (S. Xu et al., 2025). A central theoretical framework organizing this literature is academic socialization theory.

Academic socialization refers to the process by which individuals acquire the knowledge, skills, values, attitudes, and norms enabling the effective performance of scholarly roles (Weidman et al., 2001). This developmental process occurs through both formal mechanisms—such as coursework, qualifying examinations, and research training—and informal interactions with faculty, peers, and other members of disciplinary communities (Austin, 2002; Gardner, 2010). Weidman’s framework identifies four interactive stages of socialization: the anticipatory stage, where students develop initial expectations; the formal stage, involving interaction with courses, faculty, and peers; the informal stage, characterized by exchange and communication for professional and personal goals; and the personal stage of self-identity development (Weidman et al., 2001).

Recent empirical studies have applied this framework across diverse contexts. Khalid et al. (2023) examined doctoral students’ research socialization in Pakistani universities, finding that students hold high expectations for research skill development but often encounter challenging learning environments. Asadullah et al. (2023) investigated “herding” behaviors in doctoral education, revealing how students navigate collective decision-making processes during their training. These studies underscore that socialization experiences vary significantly across institutional and cultural contexts, highlighting the need for context-sensitive research.

### 2.2. Identity, Belonging, and Professional Community

Central to academic socialization is the construction of scholarly identity—an evolving sense of self as a legitimate member of an academic community with particular expertise, commitments, and ways of engaging with knowledge (Mantai, 2017). Professional identity formation (PIF) has emerged as a distinct research domain, with scholars examining how students develop, negotiate, and transform their professional self-understandings throughout graduate training (Reissner & Armitage-Chan, 2024; Tomlinson & Jackson, 2021).

Identity development involves not only acquiring technical competencies but also internalizing disciplinary values, understanding tacit norms of scholarly practice, and developing a sense of belonging within professional communities. These processes unfold gradually through iterative experiences of legitimate peripheral participation, wherein newcomers progressively assume more central roles in community practices (Lave & Wenger, 1991).

A growing body of research emphasizes the importance of belonging and community connection for graduate student success and well-being. Belonging has been identified as a critical factor affecting students’ academic engagement, motivation, persistence, and overall achievement (Allen et al., 2024; Strayhorn, 2018). For doctoral students specifically, the development of a sense of belonging within scholarly communities is closely linked to successful completion and positive career outcomes.

Professional networks have been shown to play crucial roles in career development, with networking behaviors significantly predicting career success across academic career stages (Heffernan, 2021; Porter et al., 2023). Academic conferences serve as key venues for building such networks, offering structured opportunities for interaction that might not otherwise occur in the relative isolation of doctoral study.

Academic conferences represent particularly significant sites for identity development because they compress multiple developmental opportunities into intensive, time-bounded experiences. Within conference settings, graduate students can observe senior scholars modeling disciplinary practices, present their own work for peer evaluation, engage in knowledge exchange across institutional boundaries, and participate in the rituals and social activities that constitute community membership (Henderson, 2019). The public nature of conference presentations, combined with evaluative audiences, creates high-stakes contexts that accelerate identity work and crystallize emerging self-understanding as scholars.

### 2.3. Academic Pressure, Mental Health, and Alienation

Contemporary graduate education unfolds within an increasingly competitive and performance-driven environment. The “publish or perish” culture that pervades academia creates particular challenges for graduate students who must simultaneously develop research competencies and demonstrate productivity (Grimes et al., 2018; Heffernan, 2021). Systematic reviews and meta-analyses indicate that mental health problems are relatively common among doctoral students. Approximately one-third to 40% of students report experiencing symptoms of depression or anxiety during their training period, and in some studies, this proportion approaches 50% (Satinsky et al., 2021; Evans et al., 2018; Levecque et al., 2017). Poor mental well-being has been identified as a major contributor to decisions to leave academia (SenthilKumar et al., 2023; Woolston, 2022).

The concept of academic alienation provides a theoretical lens for understanding these challenges beyond individual mental health symptoms. Academic alienation refers to states of estrangement from scholarly work, collegial relationships, or disciplinary communities that may arise when structural conditions undermine the intrinsic values and relational foundations of academic practice (Gardner & Mendoza, 2023; Vekkaila et al., 2013). Building on Marxian conceptions of alienated labor and subsequent applications in educational contexts, academic alienation can manifest in multiple dimensions: alienation from the products of scholarly work; alienation from the process of inquiry; alienation from fellow scholars; and alienation from one’s scholarly self (Jazvac-Martek et al., 2011).

Bello et al. (2023) examined how publish-or-perish culture creates reputation disparities that affect both research productivity and teaching, suggesting that alienation dynamics operate across multiple dimensions of academic work. These studies collectively indicate that alienation experiences extend beyond individual psychological distress to encompass broader disruptions in scholarly meaning-making and professional identity development.

### 2.4. Academic Conference Participation: Current Understanding and Gaps

Despite the recognized importance of conferences in scholarly life, systematic research on graduate students’ conference experiences remains limited. Existing studies have primarily adopted institutional or quantitative perspectives, examining participation rates, funding mechanisms, or organizational factors (Rowe, 2018; Henderson, 2019). Nicolson (2017) critically examined conferences as neoliberal commodities, highlighting how marketization pressures shape conference experiences. However, less attention has been devoted to understanding how graduate students themselves experience conference engagement, what motivates their participation, and how these experiences contribute to their evolving academic identities.

This gap is significant because conferences offer unique affordances for academic socialization that differ from other developmental contexts. Unlike the hierarchical dynamics of advisor–student relationships or the solitary nature of dissertation writing, conferences situate graduate students within a broader scholarly community where they can observe disciplinary norms, receive feedback on their work, build professional networks, and envision future career trajectories (Shin et al., 2018). The intensive, time-bounded nature of conference experiences creates conditions for accelerated identity work that merit empirical investigation.

### 2.5. Theoretical Integration and Research Framework

We integrate the theoretical perspectives reviewed above to conceptualize conference participation as a potentially transformative practice occurring at the intersection of socialization processes, alienation dynamics, and community building. Our integrative framework draws on three interconnected theoretical traditions that together illuminate the complexity of graduate students’ conference experiences.

First, Weidman’s academic socialization framework (Weidman et al., 2001) provides the foundational structure for understanding how conferences contribute to knowledge acquisition, skill development, and identity formation. This framework’s four-stage model—anticipatory, formal, informal, and personal—helps explain how students progress from initial expectations about conference participation through structured engagement to deeper identity integration. Within this framework, conferences serve as concentrated sites where multiple socialization mechanisms operate simultaneously, accelerating developmental processes that might otherwise unfold more gradually.

Second, the community of practice perspective (Lave & Wenger, 1991) illuminates how conference participation enables legitimate peripheral participation, wherein newcomers gradually move from peripheral observation to central contribution within scholarly communities. This theoretical lens helps explain the developmental trajectory we anticipate observing, from exploratory attendance through active presentation to community leadership. The concept of legitimate peripheral participation is particularly relevant to conference settings, where hierarchies are simultaneously visible and temporarily flattened through shared participation in scholarly rituals.

Third, alienation theory, adapted from Marxian conceptions and applied to educational contexts (Gardner & Mendoza, 2023; Jazvac-Martek et al., 2011; Vekkaila et al., 2013), provides a critical lens for examining how contemporary academic pressures create conditions of estrangement from scholarly work, relationships, and communities. This perspective suggests that conference participation may serve not merely developmental functions but also restorative ones, offering spaces where the intrinsic values of scholarship become visible and recoverable.

The integration of these frameworks generates several propositions that guide our inquiry. We anticipate that students will exhibit diverse orientations to conference participation, reflecting different developmental priorities and socialization needs. We expect that alienation experiences in daily academic work will contextualize conference participation, making such experiences particularly meaningful for students experiencing disconnection. Additionally, we hypothesize that successful conference participation may produce transformative outcomes that counteract alienation and advance identity development.

**Figure 1:**
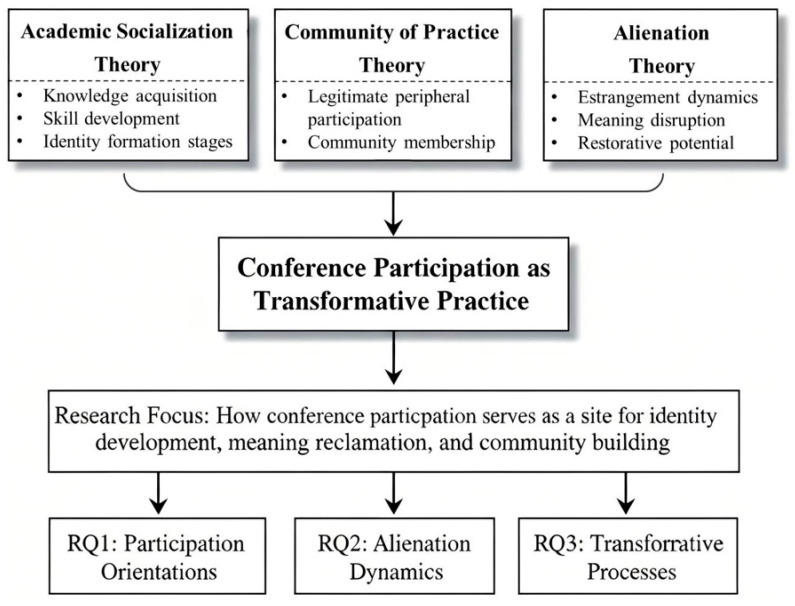
Integrative Theoretical Framework for Understanding Graduate Students’ Conference Participation.

Figure 1 provides a visual representation of this integrative theoretical framework, illustrating how the three theoretical traditions converge to inform our research focus and guide our three research questions.

**Figure 2:**
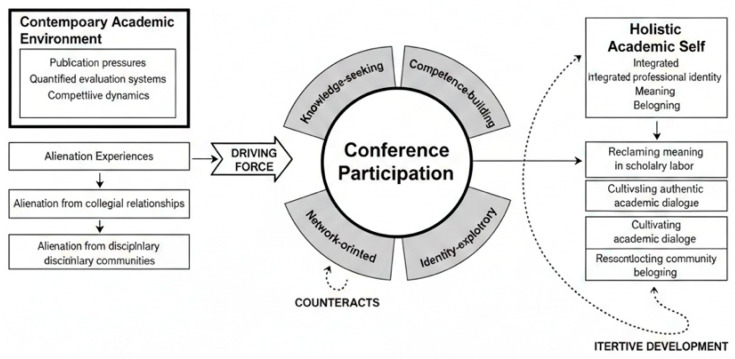
Conceptual Framework: Conference Participation as Transformative Practice in Graduate Student Academic Socialization.

This framework positions student agency as central, recognizing that graduate students actively interpret and shape their conference experiences rather than passively receiving socialization. The theoretical integration depicted in Figure 1 provides the foundation for our empirical investigation and will be elaborated with empirical findings in the conceptual model presented in the Section 5 (Figure 2).

## 3. Methods

### 3.1. Research Design

This study employed a qualitative research design grounded in interpretive phenomenology (Smith et al., 2021). This approach was selected for its capacity to illuminate the participants’ lived experiences and the meanings they ascribe to their engagement with academic conferences. Interpretive phenomenology emphasizes understanding phenomena from the perspective of those who experience them while acknowledging the active role of the researcher in interpreting and representing these experiences. While informed by interpretive phenomenological commitments to understanding lived experience, this study employed reflexive thematic analysis (Braun & Clarke, 2022) as a flexible analytic strategy compatible with phenomenological orientations yet suitable for examining patterns across a larger participant sample.

### 3.2. Participants

Participants were recruited through purposive sampling supplemented by snowball referrals, seeking variation in disciplinary background, degree level, gender, and conference experience. Inclusion criteria specified current enrollment in a graduate program (master’s or doctoral level) at a Chinese university, with at least one prior experience attending an academic conference.

The final sample comprised 18 graduate students representing diverse fields: humanities and social sciences (*n* = 10), natural sciences and engineering (*n* = 6), and medical and health sciences (*n* = 2). Doctoral students constituted the majority of the sample (*n* = 11), with the remainder pursuing master’s degrees (*n* = 7). Participants ranged from first-year master’s students to advanced doctoral candidates, with conference experience varying from single attendance to extensive participation including international presentations. The sample included more female participants (*n* = 12) than male participants (*n* = 6). Table 1 provides detailed participant characteristics.

### 3.3. Data Collection

Data were collected through semi-structured interviews conducted between September 2024 and May 2025. An interview protocol was developed based on the research questions and theoretical framework, covering the following domains: (a) motivations for conference participation; (b) descriptions of significant conference experiences; (c) challenges encountered during participation; (d) perceived impacts on research and professional development; and (e) reflections on the relationship between conference experiences and broader academic environment.

Interviews were conducted in Mandarin Chinese by trained researchers, either in person or via video conferencing according to participant preference. Sessions lasted 60–90 min and were audio-recorded with participant consent. Recordings were transcribed verbatim, producing approximately 320 pages of text. All identifying information was removed, and participants were assigned pseudonymous identifiers.

### 3.4. Data Analysis

Analysis followed the principles of reflexive thematic analysis as developed by Braun and Clarke (2023), proceeding through iterative phases of familiarization, coding, and theme development. Reflexive thematic analysis positions researcher subjectivity as a resource rather than a threat to be contained, emphasizing that meaning and knowledge are contextually situated, partial, and provisional (Braun & Clarke, 2023). This approach aligns with our interpretivist orientation and enables rich, nuanced engagement with participants’ experiences.

Initial coding was conducted openly, identifying discrete meaning units within transcripts. Through progressive abstraction and comparison, related codes were clustered into candidate themes, which were subsequently refined through team discussion and re-examination of source data. Consistent with reflexive thematic analysis principles, we treated themes as interpretive constructions generated through active analytic engagement rather than as entities “emerging” passively from the data.

Analysis was supported by NVivo 12 Plus (QSR International, Melbourne, Australia), facilitating systematic organization of codes and themes. To enhance trustworthiness, we employed multiple strategies: investigator triangulation (independent coding by two researchers with subsequent reconciliation); member checking (sharing preliminary findings with a subset of participants for validation); and reflexive journaling (documenting analytical decisions and researcher positionality throughout the process) (Lincoln & Guba, 1985; Tracy, 2010).

### 3.5. Ethical Considerations

This study received ethical approval from the Ethics Review Committee of the Educational Science Research Institution in the region where the first author is based. All participants provided written informed consent prior to data collection, with assurances of confidentiality and the right to withdraw without any consequences. Anonymous identifiers were used throughout the study to protect the participants’ identities.

## 4. Findings

Our analysis identified three overarching themes that illuminate graduate students’ conference participation experiences: (1) diverse orientations to participation, reflecting varied motivations and engagement patterns; (2) the presence of alienation dynamics in contemporary academic environments that contextualize participation; and (3) transformative processes through which conference participation supports identity development and counters alienation. We present each theme with supporting evidence from participant accounts.

### 4.1. Orientations to Conference Participation

Analysis revealed four distinct orientations that characterized the participants’ approaches to conference engagement. While individuals often exhibited elements of multiple orientations, most participants showed a predominant pattern reflecting their primary motivations and values. These orientations were observed across disciplinary backgrounds, though some patterns emerged regarding program stage, as discussed below.

#### 4.1.1. Knowledge-Seeking Orientation

Participants exhibiting a knowledge-seeking orientation prioritized acquiring current disciplinary knowledge and expanding their intellectual horizons. These individuals approached conferences as efficient platforms for accessing frontier research, novel methodologies, and emerging theoretical perspectives. As one participant explained:

*“My main purpose for attending conferences is to understand the latest developments in my field—to see what others are working on, what new methods and theories are emerging. This is incredibly valuable for informing my own research direction.”*

*(P5, doctoral student, year 2, social sciences, female)*

Knowledge-seekers demonstrated systematic engagement behaviors: carefully reviewing conference programs in advance, strategically selecting sessions aligned with their research interests, taking detailed notes during presentations, and subsequently integrating acquired insights with their ongoing work. A participant from medical sciences described her practice:

*“For every conference, I bring a dedicated notebook. I record key points, methodological approaches, anything potentially useful. Afterward, I transcribe and annotate these notes, marking sections relevant to my work. This has become my standard routine.”*

*(P11, master’s student, year 1, medical sciences, female)*

These participants evaluated conferences primarily by their intellectual content and relevance, gravitating toward flagship disciplinary meetings and specialized symposia addressing their research domains. The knowledge-seeking orientation was prominent across all disciplines and program stages, though it was particularly emphasized among participants in the natural sciences and engineering fields.

#### 4.1.2. Competence-Building Orientation

Competence-building participants viewed conferences as developmental arenas for enhancing scholarly capabilities and validating their research through peer evaluation. The opportunity to present work publicly, receive critical feedback, and refine academic communication skills motivated their engagement. As one participant articulated:

*“Presenting at conferences represents both a challenge and a growth opportunity for me. Preparing forces me to clarify my thinking and sharpen my expression. Responding to questions reveals blind spots in my work that I couldn’t see before.”*

*(P3, doctoral student, year 2, social sciences, female)*

These individuals invested substantially in presentation preparation, actively sought feedback during and after sessions, and systematically incorporated critiques into subsequent research iterations. They conceptualized conferences as “testing grounds” for assessing their scholarly standing relative to peers. One participant from natural sciences recounted a formative experience:

*“At one conference, a senior professor offered sharp criticism of my methodology. I was initially devastated, but upon reflection, I recognized the validity of his points. That painful experience proved crucial for my development as a researcher.”*

*(P15, doctoral student, year 2, natural sciences, male)*

The competence-building orientation was particularly prominent among doctoral students in their second and third years, who were actively developing their independent research profiles. Both male and female participants expressed this orientation, though female participants more frequently mentioned anxiety about public speaking alongside their growth motivation.

#### 4.1.3. Network-Oriented Participation

Network-oriented participants emphasized the relational dimensions of conference engagement, viewing these events primarily as opportunities for building professional connections, entering disciplinary communities, and accessing career-relevant resources. A social sciences doctoral student explained:

*“For me, the greatest value of conferences lies in meeting colleagues—especially those working on similar topics. Through conferences, I’ve met several key collaborators whose connections have significantly benefited both my research and career development.”*

*(P6, doctoral student, year 2, social sciences, female)*

These participants demonstrated pronounced social engagement: actively participating in informal gatherings, strategically initiating conversations with targeted individuals, and systematically maintaining relationships established at conferences. They recognized networking as requiring ongoing investment beyond the conference itself:

*“I pay special attention to social events—coffee breaks, dinners, site visits. These seemingly informal moments often yield unexpected opportunities. At one lunch session, I met someone who later became very important to my job search.”*

*(P14, master’s student, year 3, natural sciences, female)*

Network-oriented participation was observed across all program stages but was most strongly expressed by students with international conference experience, who recognized the distinctive value of cross-institutional connections. Interestingly, this orientation showed no clear gender pattern in our sample, with both male and female participants equally emphasizing relational engagement.

#### 4.1.4. Identity-Exploratory Orientation

Participants with an identity-exploratory orientation approached conferences as opportunities for investigating the nature of academic life and clarifying their own professional aspirations. Rather than pursuing specific instrumental outcomes, these individuals sought to experience scholarly culture, observe professional role models, and assess their fit with academic career paths. One doctoral student described:

*“When I first started my PhD, I attended conferences without any specific goals. At that time, I simply wanted to experience what academic life was really like and to see whether this was the kind of life and career I wanted. This kind of experiential learning cannot be gained from the literature.”*

*(P8, doctoral student, year 4, social sciences, male)*

Identity-exploratory participants demonstrated openness to diverse conference experiences, attended to tacit dimensions of scholarly practice, and used observations to inform self-reflection about career directions:

*“By attending conferences, I can observe how different scholars ask questions, debate, and express themselves. These details reveal the values and norms of the academic community. They help me consider whether I belong in this world.”*

*(P13, master’s student, year 2, engineering, female)*

This orientation was most pronounced among early-stage students (first and second year) and among those expressing uncertainty about pursuing academic careers. Students in applied fields (engineering, medical sciences) were more likely to express this exploratory stance, perhaps reflecting the availability of non-academic career alternatives in these disciplines.

#### 4.1.5. Dynamic Interplay Among Orientations

While these orientations represent analytically distinct patterns, participants typically exhibited hybrid configurations that evolved over their graduate careers. Early-stage students often emphasized identity-exploratory dimensions, while more advanced students increasingly integrated competence-building and network-oriented elements. One participant reflected on this developmental trajectory:

*“When I first started attending conferences, I was just observing and absorbing—trying to understand this world. Now I present, engage in discussions, and actively build relationships. My purposes have evolved as I’ve progressed.”*

*(P10, doctoral student, year 4, social sciences, female)*

Analysis across participant characteristics revealed several patterns. Doctoral students more frequently exhibited competence-building and network-oriented patterns compared to master’s students, who more often emphasized knowledge-seeking. Students in humanities and social sciences showed slightly stronger network orientation than those in natural sciences and engineering, possibly reflecting disciplinary differences in collaboration norms. Female participants across disciplines more frequently mentioned emotional dimensions of conference participation, including both anxiety and connection, though orientation patterns were otherwise similar across genders.

Themes of seeking academic community and overcoming isolation appeared consistently across all participant subgroups, suggesting that these may represent shared experiences transcending disciplinary and demographic differences. This dynamic quality underscores the developmental nature of conference participation and its embeddedness in broader socialization trajectories.

### 4.2. Alienation Dynamics in Contemporary Academia

Beneath the participants’ specific conference experiences, we identified a deeper thematic layer concerning the challenging conditions of contemporary academic environments. Participants described feeling alienated from their scholarly work, collegial relationships, and disciplinary communities—conditions that both contextualized their conference participation and were partially addressed through it. These alienation experiences were remarkably consistent across disciplinary fields, program stages, and genders, suggesting systemic rather than individual origins.

#### 4.2.1. Alienation from Scholarly Labor

Participants widely reported experiencing disconnection between their daily research activities and the intrinsic values of scholarly inquiry. Quantified evaluation systems, publication pressures, and routinized tasks contributed to a sense that research had become instrumentalized—valued primarily for measurable outputs rather than intellectual contribution. One humanities student expressed:

*“My current research feels like assembly-line work: writing my dissertation, producing small papers, meeting performance indicators, and completing required forms. At times, I ask myself: what is the significance of the research I am doing? Am I really pursuing truth?”*

*(P9, doctoral student, year 4, humanities, female)*

Participants described their daily work as fragmented into discrete, disconnected tasks that obscured larger intellectual purposes. This fragmentation impaired their ability to perceive meaning in individual activities and contributed to emotional exhaustion:

*“Much of my time is spent working for my supervisor—helping prepare presentation slides, drafting documents, and handling related tasks. I have little space to think about broader questions or to develop my own research ideas. This situation leaves me feeling empty and exhausted.”*

*(P2, master’s student, year 2, humanities, female)*

#### 4.2.2. Alienation from Collegial Relationships

Participants also described challenges in their academic relationships, characterized by superficiality, instrumentalism, and limited authentic exchange. Competitive pressures appeared to undermine collaborative norms, replacing genuine intellectual engagement with transactional interactions:

*“In my department, people rarely share their actual thoughts about research. Everyone protects their ideas, worrying about being scooped. Conversations stay at the surface level—polite but not genuinely engaging.”*

*(P4, doctoral student, year 2, social sciences, female)*

This relational alienation extended to faculty interactions, where hierarchical dynamics sometimes impeded authentic mentorship:

*“I feel like my advisor and I don’t really communicate about ideas. My supervisor is like a boss. Our meetings are about checking progress boxes rather than discussing intellectual questions. I crave substantive dialogue but rarely find it.”*

*(P17, doctoral student, year 3, computer science, male)*

Several participants connected these patterns to broader environmental factors, including competitive funding structures, time pressures, and evaluation systems that rewarded individual achievement over collaborative engagement.

#### 4.2.3. Alienation from Disciplinary Communities

Beyond interpersonal relationships, participants described feeling disconnected from broader scholarly communities—uncertain about disciplinary norms, unclear about their place within the field, and lacking a sense of belonging. Graduate education, concentrated within single institutions and often small research groups, sometimes left students feeling isolated from wider disciplinary discourse:

*“My daily work happens in a small circle—my lab, my advisor, a few classmates. But I have little sense of the broader field: who the key players are, what the major debates involve, where my work fits. This isolation creates constant uncertainty about whether I’m on the right track.”*

*(P12, master’s student, year 2, engineering, male)*

This community alienation contributed to identity ambiguity, as students lacked reference points for evaluating their progress and positioning themselves within professional landscapes.

### 4.3. Conference Participation as Transformative Practice

Against this backdrop of alienation, conference participation emerged as a significant counterweight—a practice through which students reclaimed meaning, connection, and identity. Three interrelated transformative processes characterized how conferences supported development.

#### 4.3.1. Reclaiming Meaning in Scholarly Labor

Conference participation provided opportunities to reconnect with the intrinsic values and broader significance of research work. Temporarily removed from the routinized demands of daily work, students encountered scholarship as living intellectual exchange rather than metric-driven production:

*“At the conference, I was able to see the bigger picture, including how my work connects to the broader field and why it matters. This helped me rediscover the passion and motivation I felt when I first set out to study medicine.”*

*(P11, master’s student, year 1, medical sciences, female)*

This meaning reclamation operated through several mechanisms. Encountering diverse research presentations helped students situate their own work within larger disciplinary conversations, clarifying potential contributions. Receiving positive feedback validated the value of their efforts. Observing scholars who embodied genuine intellectual curiosity reminded participants of academic ideals that daily pressures sometimes obscured.

Participants described reclaiming meaning on multiple levels: redefining research goals from external evaluation standards toward internal academic value; reconsidering research methods to prioritize fit with research questions rather than technical sophistication; and reconceptualizing how to present research contributions with clarity rather than jargon:

*“Through hearing different presentation styles, I realized that good research isn’t about showing off complex methods and technical terms. It’s about clearly expressing core ideas and contributions. This changed how I write papers and give presentations.”*

*(P7, doctoral student, year 3, social sciences, female)*

Significantly, participants described translating conference insights into transformed orientations toward their regular work:

*“After conferences, I’ve started reserving time each week for reading beyond my immediate project—thinking about bigger questions. This has expanded my perspective and made daily work feel more meaningful.”*

*(P15, doctoral student, year 2, natural sciences, male)*

#### 4.3.2. Cultivating Authentic Academic Dialogue

Conferences also provided contexts for developing more genuine, substantive modes of academic communication. Through observing skilled scholarly exchange and practicing their own engagement, participants developed enhanced capacities for authentic dialogue:

*“At first, I attended academic conferences mainly to visit places I hadn’t been to and do a bit of sightseeing. Gradually, I realized that conferences broaden my horizons and let me hear scholars’ perspectives collide.”*

*(P16, doctoral student, year 2, engineering, male)*

This development involved acquiring both technical skills—clear presentation, constructive questioning, gracious response to criticism—and deeper orientations toward dialogue as collaborative meaning-making rather than competitive display:

*“At first, when I gave presentations, I just read straight from a script; now I can speak extemporaneously.”*

*(P8, doctoral student, year 4, social sciences, male)*

Participants noted that conference experiences reshaped their approach to academic communication more broadly, influencing how they engaged in seminars, with collaborators, and in written scholarship. The cultivation of authentic dialogue countered the superficial, instrumental communication patterns that characterized everyday academic interactions in competitive environments.

#### 4.3.3. Reconstructing Community Belonging

Perhaps most significantly, conference participation contributed to developing a sense of belonging within scholarly communities. Through repeated engagement, students progressed from peripheral observers to recognized participants in disciplinary discourse:

*“Through attending conference after conference, I’ve moved from outsider to insider. I now know who’s doing what research, how different schools of thought debate, where I stand in this network. This belonging has significantly reduced my anxiety about my place in academia.”*

*(P13, master’s student, year 2, engineering, female)*

Community reconstruction operated across cognitive, affective, and behavioral dimensions. Cognitively, conferences provided maps of disciplinary landscapes—key figures, active debates, research trajectories. Affectively, collective participation in scholarly rituals fostered emotional connection and belonging. Behaviorally, participants gradually transitioned from passive attendance to active contribution, eventually organizing sessions and mentoring newer students:

*“Our field doesn’t have many academic conferences. A while ago, I volunteered at one conference, and I also learned a lot of cutting-edge ideas in sports training.”*

*(P18, master’s student, year 3, sports science, male)*

Such accounts suggest that conference participation can support development from passive socialization recipient to active community member—a transition from being shaped by academic culture to participating in its construction. This progression involves not merely joining existing communities but actively contributing to more inclusive and supportive scholarly networks:

*“As young scholars, we need to both respect disciplinary traditions and bring new perspectives. Conferences give us opportunities to learn from senior scholars while also contributing our unique insights.”*

*(P1, master’s student, year 1, humanities, female)*

### 4.4. Essence of the Conference Participation Experience

Across the diverse accounts shared by our participants, a unifying phenomenological essence emerged that captures the fundamental meaning of conference participation for graduate students navigating contemporary academic environments. At its core, the conference experience represents a transformative liminal space—a temporary yet consequential departure from the fragmented, pressurized routines of daily academic life into an arena where the deeper values of scholarship become visible, tangible, and recoverable.

Participants consistently described conferences as sites where they could momentarily step outside the instrumentalizing pressures of metrics, deadlines, and evaluations to reconnect with the intrinsic purposes that initially drew them to scholarly work. This reconnection was not merely cognitive but deeply felt—an emotional recalibration that reminded them why their work matters and who they aspire to become as scholars. The experience transcended individual sessions or presentations; it resided in the totality of immersion in scholarly community: the animated hallway conversations, the recognition of shared struggles with fellow students, the inspiring encounters with scholars who embodied intellectual passion, and the gradual realization that one belongs in this world.

What emerged most powerfully across participant narratives was the experience of conferences as spaces of authentic connection in environments otherwise characterized by isolation and competition. Participants found at conferences what they often lacked in their daily academic lives: genuine intellectual dialogue unburdened by evaluation anxiety, relationships formed around shared curiosity rather than strategic calculation, and a sense of participating in something larger than their individual projects. For students experiencing alienation from their work, their peers, and their disciplinary communities, conferences offered fleeting but meaningful experiences of wholeness—moments when the fragmented pieces of academic identity cohered into an integrated sense of scholarly self.

This essence illuminates why conference participation held such significance for participants despite its time-bounded nature. Conferences functioned not as escapes from academic reality but as encounters with academic ideals—reminders of what scholarly life could and should be, and sources of motivation and meaning that participants carried back into their daily work. The transformative potential of these experiences lay precisely in this capacity to restore the participants’ relationship with the deeper purposes of their vocation.

## 5. Discussion

This study illuminates how graduate students engage with academic conferences as developmental sites within contemporary academic environments. Our findings advance the understanding of academic socialization by revealing both the diverse orientations students bring to conference participation and the transformative potential of these experiences for addressing alienation and constructing scholarly identity.

### 5.1. Diverse Orientations and Developmental Trajectories

The four participation orientations we identified—knowledge-seeking, competence-building, network-oriented, and identity-exploratory—reflect the multifaceted nature of conference experiences and the varied developmental needs of graduate students. These orientations parallel dimensions of academic socialization previously identified in the literature, including knowledge acquisition, skill development, network building, and identity formation (Gardner & Mendoza, 2023; Weidman et al., 2001). However, our findings extend prior work by demonstrating how these dimensions manifest in the specific context of conference participation and how they dynamically evolve across graduate careers.

The developmental trajectory we observed—from exploratory observation through active engagement to community contribution—aligns with models of legitimate peripheral participation in communities of practice (Lave & Wenger, 1991) and with Weidman’s stages of socialization (Khalid et al., 2023). Early-career students appropriately emphasized observation and meaning-making, while advanced students increasingly integrated competence demonstration and relational development. This progression suggests that effective support for conference participation should be developmentally calibrated, with different emphases and interventions appropriate for different career stages. Our finding that doctoral students more frequently exhibited competence-building and network-oriented patterns resonates with research documenting how doctoral education progressively emphasizes independent scholarly identity (Mantai, 2017).

Our findings also resonate with recent research on professional identity formation, which emphasizes the iterative, contextually embedded nature of identity development. The hybrid and evolving nature of participation orientations suggests that identity construction occurs through ongoing negotiation rather than linear progression, with conferences serving as occasions for identity experimentation and consolidation.

### 5.2. Alienation and the Contemporary Academic Environment

A significant contribution of this study lies in connecting conference participation to broader dynamics of academic alienation. Participants’ accounts of disconnection from scholarly labor, collegial relationships, and disciplinary communities resonate with growing concerns about graduate student well-being and the impact of performance-driven academic cultures (Castelló et al., 2017; Vekkaila et al., 2013). The pervasive publication pressures, quantified evaluation systems, and competitive dynamics described by the participants create conditions that can undermine intrinsic motivation, authentic relationships, and community belonging. Notably, these alienation experiences appeared remarkably consistent across our diverse sample, suggesting systemic rather than discipline-specific or individual-level causes—a finding that aligns with structural analyses of contemporary academic labor conditions (Bello et al., 2023).

These alienation dynamics are particularly concerning given evidence linking academic pressure to mental health challenges. Meta-analyses indicate elevated rates of depression, anxiety, and suicidal ideation among doctoral students compared to the general population (Evans et al., 2018; Levecque et al., 2017; Satinsky et al., 2021; W. Xu et al., 2024). Our findings suggest that alienation experiences extend beyond mental health symptoms to encompass broader disruptions in scholarly meaning-making and professional identity development. Research on doctoral student well-being has similarly emphasized how structural conditions shape psychological experiences (Woolston, 2022), and our findings contribute to this literature by documenting specific mechanisms through which alienation manifests and may be addressed.

Our findings suggest that these alienation dynamics importantly contextualize conference participation. Students are not merely seeking developmental opportunities in a neutral environment; they are navigating challenges that shape their relationship to scholarly work itself. Understanding conference participation within this context illuminates both why such experiences hold significance for students and what specific functions they may serve.

### 5.3. The Transformative Role of Conference Participation

The transformative processes we identified—reclaiming scholarly meaning, cultivating authentic dialogue, and reconstructing community belonging—represent ways that conference participation can counteract alienation and support positive identity development. These findings extend prior conceptions of conference functions beyond knowledge transfer and networking to encompass deeper identity work and meaning-making. Whereas previous research has emphasized instrumental conference benefits such as presentation opportunities and career networking (Henderson, 2019; Rowe, 2018), our findings reveal more fundamental identity and meaning-restoration functions that may be particularly crucial for students experiencing alienation.

Particularly notable is the agentic quality of the participants’ engagement. Rather than passive recipients of socialization, students actively interpreted experiences, extracted lessons, and translated insights into transformed practices. This agency aligns with contemporary understandings of socialization as an interactive process wherein individuals both internalize community norms and actively shape their own development. Malinowski et al. (2024) documented similar patterns of agentic socialization among doctoral students, finding that students actively construct meaning from their educational experiences rather than passively absorbing institutional norms.

The finding that conference experiences can help students “reclaim” meaning in scholarly labor has practical implications. It suggests that periodic immersion in settings that foreground intellectual exchange—removed from daily production pressures—may serve restorative functions for graduate students experiencing alienation. This interpretation aligns with research on virtual communities of practice, which emphasizes how scholarly communities can provide mutual support and counteract isolation. Our finding that participants translated conference insights into sustained changes in daily practice further suggests that these transformative effects extend beyond the conference setting itself, creating positive spillover effects into regular academic work.

### 5.4. An Integrative Conceptual Framework

Building on our empirical findings, we propose an integrative conceptual framework that synthesizes the relationships among alienation dynamics, participation orientations, conference experiences, and transformative outcomes (see Figure 2). This framework extends the theoretical foundation presented in Figure 1 by incorporating our empirical findings to position academic conference participation as a pivotal mediating practice within the broader landscape of graduate student socialization.

The framework illustrates several key dynamics. First, the contemporary academic environment, characterized by publication pressures and quantified evaluation systems, generates alienation experiences that paradoxically serve as drivers of conference participation. Students seek conferences partly as refuges from the fragmenting pressures of daily academic work, spaces where the intrinsic values of scholarship remain visible and celebrated. This dynamic resonates with Vekkaila et al.’s (2013) findings that doctoral students experiencing disengagement seek alternative contexts for reconnection with scholarly purpose.

Second, students approach conferences with distinct orientations—knowledge-seeking, competence-building, network-oriented, and identity-exploratory—that shape how they engage with conference opportunities. These orientations are not static; they evolve across students’ academic careers, typically progressing from exploratory observation toward more active, multifaceted engagement. The framework captures this developmental dynamism through feedback loops connecting transformative outcomes back to participation orientations. This progression aligns with Weidman et al.’s (2001) four-stage socialization model, wherein students move from anticipatory expectations through formal and informal engagement to personal identity integration.

Third, and most centrally, Figure 2 positions conference participation as a transformative site where students can counteract alienation through three interrelated processes: reclaiming meaning in scholarly labor, cultivating authentic academic dialogue, and reconstructing community belonging. These processes operate synergistically, each reinforcing the others in supporting students’ development toward what we term the “holistic academic self”—an integrated professional identity that balances productivity with meaning, competition with community, and individual achievement with collegial belonging. This conceptualization extends Mantai’s (2017) work on “feeling like a researcher” by specifying the mechanisms through which such feelings develop and are sustained.

The framework’s feedback loops highlight the recursive nature of these dynamics. Successful transformation through conference participation may reduce alienation experiences in daily academic work while simultaneously enabling students to engage with subsequent conferences from more advanced participation orientations. This recursive quality suggests that conference participation can initiate virtuous cycles of professional development, with each positive experience building capacity for deeper engagement in future scholarly activities. Conversely, negative conference experiences—such as dismissive feedback or failed networking attempts—might reinforce alienation and discourage future engagement, a dynamic that underscores the importance of supportive conference environments.

Importantly, the framework acknowledges that transformation is not automatic. Students’ capacity to benefit from conference participation depends on multiple factors, including the quality of conference experiences themselves, the support provided by mentors and institutions, and students’ own reflective engagement with their experiences. Gillani et al.’s (2023) review of doctoral mentorship similarly emphasizes how structural supports shape the developmental potential of socialization experiences. The framework thus implies intervention points where stakeholders can enhance the transformative potential of conference participation.

### 5.5. Implications for Practice

Our findings suggest several implications for supporting graduate student development through conference participation, each grounded in the empirical patterns documented in this study:

For institutions: Results underscore the importance of supporting graduate student conference participation not merely through funding but through comprehensive preparation, mentorship, and post-conference integration. Pre-conference workshops on presentation skills and networking strategies, mentored participation for novice attendees, and structured post-conference reflection could enhance developmental outcomes. Our finding that alienation experiences were consistent across disciplines suggests that institutional-level interventions addressing systemic pressures may be more effective than discipline-specific approaches. Institutions should also consider how evaluation systems might be modified to reduce alienating pressures while maintaining appropriate standards. Research on institutional supports for doctoral students (Khalid et al., 2023; Malinowski et al., 2024) provides additional guidance for designing comprehensive support structures.

For faculty advisors: Findings highlight opportunities to leverage conference experiences as developmental interventions, particularly for students showing signs of alienation or identity uncertainty. Our finding that alienation from scholarly labor often manifested as feeling disconnected from intrinsic research values suggests that advisors might use conference preparation as an occasion for discussing the broader significance of the students’ work. Advisors can help students identify conferences aligned with their developmental needs, provide guidance on navigating conference social dynamics, and facilitate integration of conference learning into ongoing work. The diverse participation orientations we identified suggest that effective mentoring should be responsive to individual students’ developmental priorities rather than assuming uniform goals. The developmental trajectory from exploration to active contribution further suggests that advising strategies should evolve as students progress through their programs.

For graduate students: Results suggest the value of approaching conferences intentionally, with clear purposes and openness to transformative experiences. Students might benefit from reflecting on their predominant participation orientation and considering whether diversifying their approach could address unmet developmental needs. Our finding that early-stage students benefited particularly from identity-exploratory engagement suggests that novice conference-goers need not pressure themselves to network aggressively or present prematurely; observation and meaning-making represent legitimate developmental activities. The importance of informal interactions suggests attending to opportunities beyond formal presentations, as these settings often provided the participants’ most meaningful connections.

### 5.6. Limitations and Future Directions

Several limitations warrant acknowledgment. Our sample, while diverse in disciplinary background, was drawn from Chinese universities, and cultural factors may shape conference participation experiences in ways that limit generalizability to other contexts. The retrospective nature of interview data may introduce recall biases and post hoc rationalization. Additionally, we relied on self-report without observational or longitudinal data to corroborate the participants’ accounts of transformation.

Future research might productively extend this work in several directions. Comparative studies across cultural and institutional contexts could illuminate how local academic environments shape conference experiences. Longitudinal designs tracking students through multiple conference experiences could more directly capture developmental trajectories. Mixed-method approaches combining qualitative depth with quantitative assessment of outcomes could strengthen claims about the impact of conference participation on measurable developmental indicators. Investigation of virtual conference participation, which has expanded significantly in recent years, could reveal whether similar transformative dynamics occur in online settings. Additionally, research examining how specific conference features (size, format, disciplinary scope) moderate transformative potential could inform conference design and student advising.

## 6. Conclusions

This qualitative study examined graduate students’ engagement with academic conferences as sites for professional development and identity construction. We identified diverse participation orientations reflecting varied motivational patterns, documented alienation dynamics that characterize contemporary academic environments, and illuminated transformative processes through which conference participation supports identity development and counters alienation.

Our findings carry implications for multiple stakeholders. For institutions, results underscore the importance of supporting graduate student conference participation through comprehensive preparation, mentorship, and post-conference integration. For faculty advisors, findings highlight opportunities to leverage conference experiences as developmental interventions. For graduate students themselves, results suggest the value of approaching conferences intentionally, with clear purposes and openness to transformative experiences.

More broadly, this study contributes to understanding academic socialization as an agentic, contextually embedded process unfolding within challenging environmental conditions. As academic environments continue to evolve—with intensifying competition, digital transformation, and changing career landscapes—attending to the sites and practices that support graduate student development becomes increasingly consequential. Academic conferences, as concentrated spaces of community formation and identity work, merit continued scholarly attention and practical investment.

The transformative potential of conference participation offers hope that even within pressurized academic environments, opportunities exist for graduate students to reclaim meaning in their work, build authentic scholarly relationships, and develop robust professional identities. Supporting students in accessing and maximizing these opportunities represents an important avenue for improving graduate education and cultivating the next generation of scholars.

## Figures and Tables

**Table 1:** Participant Demographics.

**ID**	**Degree**	**Discipline**	**Year**	**Gender**	**Conference Type**	**Research Area**
P1	Master’s	Humanities	1	F	Domestic	Philosophy
P2	Master’s	Humanities	2	F	Domestic	Literature
P3	Doctoral	Social Sciences	2	F	Domestic/International	Education Policy
P4	Doctoral	Social Sciences	2	F	Domestic/International	Higher Education
P5	Doctoral	Social Sciences	2	F	Domestic/International	Economics
P6	Doctoral	Social Sciences	2	F	Domestic/International	Industry-Education
P7	Doctoral	Social Sciences	3	F	Domestic/International	Educational Psychology
P8	Doctoral	Social Sciences	4	M	Domestic/International	Public Policy
P9	Doctoral	Humanities	4	F	Domestic/International	Linguistics
P10	Doctoral	Social Sciences	4	F	Domestic/International	STEM Education
P11	Master’s	Medical Sciences	1	F	Domestic	Clinical Medicine
P12	Master’s	Engineering	2	M	Domestic	Integrated Circuits
P13	Master’s	Engineering	2	F	Domestic	Vehicle Engineering
P14	Master’s	Natural Sciences	3	F	Domestic	Aquatic Biology
P15	Doctoral	Natural Sciences	2	M	Domestic	Aquaculture
P16	Doctoral	Engineering	2	M	Domestic/International	Mechanical Engineering
P17	Doctoral	Engineering	3	M	Domestic	Computer Science
P18	Master’s	Sports Science	3	M	Domestic	Athletic Training

## Data Availability

The data that support the findings of this study are available from the corresponding author, Mengting Qian, upon reasonable request. The data are not publicly available due to privacy considerations.

## References

[B1-behavsci-16-00217] Allen K. A., Slaten C., Lan M., Craig H., May F., Counted V. (2024). Belonging in higher education: A twenty-year systematic review. Journal of University Teaching and Learning Practice.

[B2-behavsci-16-00217] Asadullah M. A., Mirza S., Haq M. Z. U., Yaqoob T., Hussain S. (2023). A qualitative inquiry of doctoral students’ herding in education and validation of ’herding in educational decisions’ scale. Higher Education Quarterly.

[B3-behavsci-16-00217] Austin A. E. (2002). Preparing the next generation of faculty: Graduate school as socialization to the academic career. The Journal of Higher Education.

[B4-behavsci-16-00217] Baker V. L., Pifer M. J. (2011). The role of relationships in the transition from doctoral student to independent scholar. Studies in Continuing Education.

[B5-behavsci-16-00217] Bello S. A., Azubuike F. C., Akande O. A. (2023). Reputation disparity in teaching and research productivity and rewards in the context of consequences of institutionalization of Publish or Perish culture in academia. Higher Education Quarterly.

[B6-behavsci-16-00217] Boden D., Borrego M., Newswander L. K. (2011). Student socialization in interdisciplinary doctoral education. Higher Education.

[B7-behavsci-16-00217] Braun V., Clarke V. (2022). Thematic analysis: A practical guide.

[B8-behavsci-16-00217] Braun V., Clarke V. (2023). Toward good practice in thematic analysis: Avoiding common problems and be (com) ing a knowing researcher. International Journal of Transgender Health.

[B9-behavsci-16-00217] Castelló M., Pardo M., Sala-Bubaré A., Suñé-Soler N. (2017). Why do students consider dropping out of doctoral degrees? Institutional and personal factors. Higher Education.

[B10-behavsci-16-00217] Chi T., Cheng L., Zhang Z. (2023). Global prevalence and trend of anxiety among graduate students: A systematic review and meta-analysis. Brain and Behavior.

[B11-behavsci-16-00217] Collins H., Leonard-Clarke W., Mason-Wilkes W. (2023). Scientific conferences, socialization, and the Covid-19 pandemic: A conceptual and empirical enquiry. Social Studies of Science.

[B12-behavsci-16-00217] Evans T. M., Bira L., Gastelum J. B., Weiss L. T., Vanderford N. L. (2018). Evidence for a mental health crisis in graduate education. Nature Biotechnology.

[B13-behavsci-16-00217] Gardner S. K. (2009). Student and faculty attributions of attrition in high and low-completing doctoral programs in the United States. Higher Education.

[B14-behavsci-16-00217] Gardner S. K. (2010). Faculty perspectives on doctoral student socialization in five disciplines. International Journal of Doctoral Studies.

[B15-behavsci-16-00217] Gardner S. K., Mendoza P. (2023). On becoming a scholar: Socialization and development in doctoral education.

[B16-behavsci-16-00217] Gillani B., Cohen F., Kirchgesler K., Asher Blackdeer A. (2023). Sites of possibilities: A scoping review to investigate the mentorship of marginalized social work doctoral students. Journal of Evidence-Based Social Work.

[B17-behavsci-16-00217] Grimes D. R., Bauch C. T., Ioannidis J. (2018). Modelling science trustworthiness under publish or perish pressure. Royal Society Open Science.

[B18-behavsci-16-00217] Hayes C. A., Moore J. T., Headley C. A., Berrios-Negron A. L., Lambert W. M. (2024). Unlocking the power of virtual networking for early-career researchers. eLife.

[B19-behavsci-16-00217] Heffernan T. (2021). Academic networks and career trajectory: ‘There’s no career in academia without networks’. Higher Education Research & Development.

[B20-behavsci-16-00217] Henderson E. F. (2019). Gender, definitional politics and ’live’ knowledge production: Contesting concepts at conferences.

[B21-behavsci-16-00217] Horta H., Li H., Chan S. J. (2024). Why do students pursue a doctorate in the era of the ’PhD crisis’? Evidence from Taiwan. Higher Education Quarterly.

[B22-behavsci-16-00217] Inouye K., McAlpine L. (2019). Developing academic identity: A review of the literature on doctoral writing and feedback. International Journal of Doctoral Studies.

[B23-behavsci-16-00217] Jazvac-Martek M., Chen S., McAlpine L. (2011). Tracking the doctoral student experience over time: Cultivating agency in diverse spaces. Doctoral education: Research-based strategies for doctoral students, supervisors and administrators.

[B24-behavsci-16-00217] Khalid S., Orynbek G., Lianyu C., Tadesse E. (2023). What goes around comes around: Shedding light on today’s doctoral student’s research socialization and who will be the future faculty. PLoS ONE.

[B25-behavsci-16-00217] Lave J., Wenger E. (1991). Situated learning: Legitimate peripheral participation.

[B26-behavsci-16-00217] Levecque K., Anseel F., De Beuckelaer A., Van der Heyden J., Gisle L. (2017). Work organization and mental health problems in PhD students. Research Policy.

[B27-behavsci-16-00217] Liddle A. M. (2025). Navigating liminal spaces: Identity development and professional competencies of doctoral scholar-practitioners. International Journal of Educational Management.

[B28-behavsci-16-00217] Lincoln Y. S., Guba E. G. (1985). Naturalistic inquiry.

[B29-behavsci-16-00217] Lucia B., Buhre F., Proszek J. (2025). Conference climates: International rhetoric workshop and inclusive learning practices. Rhetoric Review.

[B30-behavsci-16-00217] Malinowski P. R., Wilson W. J., Warner P. H., Trad A. M., Rifenburg P., Richards K. A. (2024). Socialization into and through doctoral programs in adapted physical activity. Adapted Physical Activity Quarterly.

[B31-behavsci-16-00217] Mantai L. (2017). Feeling like a researcher: Experiences of early doctoral students in Australia. Studies in Higher Education.

[B32-behavsci-16-00217] Nicolson D. J. (2017). Academic conferences as neoliberal commodities. Qualitative Research.

[B33-behavsci-16-00217] Porter C. M., Woo S. E., Alonso N., Snyder G. (2023). Why do people network? Professional networking motives and their implications for networking behaviors and career success. Journal of Vocational Behavior.

[B34-behavsci-16-00217] Reissner S., Armitage-Chan E. (2024). Manifestations of professional identity work: An integrative review of research in professional identity formation. Studies in Higher Education.

[B35-behavsci-16-00217] Rowe N. (2018). ‘When you get what you want, but not what you need’: The motivations, affordances and shortcomings of attending academic/scientific conferences. International Journal of Research in Education and Science.

[B36-behavsci-16-00217] Satinsky E. N., Kimura T., Kiang M. V., Abebe R., Cunningham S., Lee H., Lin X., Liu C. H., Rudan I., Sen S., Tomlinson M., Yaver M., Tsai A. C. (2021). Systematic review and meta-analysis of depression, anxiety, and suicidal ideation among Ph.D. students. Scientific Reports.

[B37-behavsci-16-00217] SenthilKumar G., Mathieu N. M., Freed J. K., Sigmund C. D., Gutterman D. D. (2023). Addressing the decline in graduate students’ mental well-being. American Journal of Physiology-Heart and Circulatory Physiology.

[B38-behavsci-16-00217] Shin J. C., Kehm B. M., Jones G. A. (2018). The increasing importance, growth, and evolution of doctoral education. Doctoral education for the knowledge society: Convergence or divergence in national approaches?.

[B39-behavsci-16-00217] Smith J. A., Flowers P., Larkin M. (2021). Interpretative phenomenological analysis: Theory, method and research.

[B40-behavsci-16-00217] Strayhorn T. L. (2018). College students’ sense of belonging: A key to educational success for all students.

[B41-behavsci-16-00217] Tomlinson M., Jackson D. (2021). Professional identity formation in contemporary higher education students. Studies in Higher Education.

[B42-behavsci-16-00217] Tracy S. J. (2010). Qualitative quality: Eight “big-tent” criteria for excellent qualitative research. Qualitative Inquiry.

[B43-behavsci-16-00217] Vekkaila J., Pyhältö K., Lonka K. (2013). Experiences of disengagement–A study of doctoral students in the behavioral sciences. International Journal of Doctoral Studies.

[B44-behavsci-16-00217] Weidman J. C., Twale D. J., Stein E. L. (2001). Socialization of graduate and professional students in higher education: A perilous passage?. ASHE-ERIC Higher Education Report.

[B45-behavsci-16-00217] Woolston C. (2022). Stress and uncertainty drag down graduate students’ satisfaction. Nature.

[B46-behavsci-16-00217] Wu Y., Chen Z., Zhang D. (2025). Supervisors’ academic supervising behaviors and graduate students’ academic thriving: The mediating roles of admiration and task engagement. Behavioral Sciences.

[B47-behavsci-16-00217] Xu S., Mansor A. N., Amat S. (2025). Higher education strategies for enhancing the employability of international students: A systematic review of the postpandemic era. Journal of International Students.

[B48-behavsci-16-00217] Xu W., Li Y., King R. B., Chen J. (2024). The well-being of doctoral students in education: An ecological systems perspective. Behavioral Sciences.

